# Green Chemistry for the Transformation of Chlorinated Wastes: Catalytic Hydrodechlorination on Pd-Ni and Pd-Fe Bimetallic Catalysts Supported on SiO_2_

**DOI:** 10.3390/gels9040275

**Published:** 2023-03-25

**Authors:** Julien G. Mahy, Thierry Delbeuck, Kim Yên Tran, Benoît Heinrichs, Stéphanie D. Lambert

**Affiliations:** 1 Department of Chemical Engineering—Nanomaterials, Catalysis & Electrochemistry, University of Liège, B6a, Quartier Agora, Allée du six Août 11, 4000 Liège, Belgium; 2 Institut National de la Recherche Scientifique (INRS), Centre-Eau Terre Environnement, Université du Québec, 490, Rue de la Couronne, Québec, QC G1K 9A9, Canada

**Keywords:** noble metal, environmental catalysis, sol–gel process, porous materials, green chemistry

## Abstract

Monometallic catalysts based on Fe, Ni and Pd, as well as bimetallic catalysts based on Fe-Pd and based on Ni-Pd supported on silica, were synthesized using a sol–gel cogelation process. These catalysts were tested in chlorobenzene hydrodechlorination at low conversion to consider a differential reactor. In all samples, the cogelation method allowed very small metallic nanoparticles of 2–3 nm to be dispersed inside the silica matrix. Nevertheless, the presence of some large particles of pure Pd was noted. The catalysts had specific surface areas between 100 and 400 m^2^/g. In view of the catalytic results obtained, the Pd-Ni catalysts are less active than the monometallic Pd catalyst (<6% of conversion) except for catalysts with a low proportion of Ni (9% of conversion) and for reaction temperatures above 240 °C. In this series of catalysts, increasing the Ni content increases the activity but leads to an amplification of the catalyst deactivation phenomenon compared to Pd alone. On the other hand, Pd-Fe catalysts are more active with a double conversion value compared to a Pd monometallic catalyst (13% vs. 6%). The difference in the results obtained for each of the catalysts in the Pd-Fe series could be explained by the greater presence of the Fe-Pd alloy in the catalyst. Fe would have a cooperative effect when associated with Pd. Although Fe is inactive alone for chlorobenzene hydrodechlorination, when Fe is coupled to another metal from the group VIIIb, such as Pd, it allows the phenomenon of Pd poisoning by HCl to be reduced.

## 1. Introduction

Over time, the presence of chlorinated organic compounds in industry increased, both in the chemical and petrochemical sectors and in the agricultural and electronic sectors. However, the discovery of the carcinogenic, mutagenic and toxic effects of these compounds led to their production being reduced and then banned. These products are nevertheless present in residual stocks and in certain chemical industry process fumes [1,2]. It is therefore necessary to develop treatment methods which allow their emissions, as well as the quantities of these compounds which have already been produced, to be eliminated.

Thermal or catalytic incineration [2,3] is the most widespread process for the treatment of these compounds because it is already in place for other wastes and is therefore well-known and simple to implement. However, the incineration of organochlorine compounds leads to the formation of other harmful compounds such as dioxins. The development of other methods is therefore necessary.

Hydrodechlorination (HDC) seems to be an interesting alternative. This process, the principle of which can be summarized by Equation (1) [4], has the advantages of producing recoverable hydrocarbons, of recovering chlorine in the form of hydrochloric acid and of not producing dioxins. This process could therefore prove to be attractive from an economic and environmental point of view [2,5].

C_x_H_y_Cl_z_ + z H_2_ → C_x_H_y+z_ + z HCl (1)

There are a wide variety of chlorinated organic compounds [6,7,8]. This work focuses on the study of the hydrodechlorination of chlorinated aromatic compounds because these are the most widespread and the most persistent. The study of the HDC reaction for this family of compounds is made through the use of a model molecule, monochlorobenzene [9,10,11], C_6_H_5_Cl, which is the simplest molecule in this family of compounds. The equation corresponding to the reaction carried out is:

C_6_H_5_Cl + H_2_ → C_6_H_6_ + HCl. (2)

This reaction must be controlled well to avoid the formation of cyclohexane. Otherwise, the need for a complex separation would arise. In addition, benzene is of much greater economic interest. High selectivity of the monochlorobenzene hydrodechlorination reaction is therefore a very important criterion in this study.

The use of a catalyst is necessary for this reaction to happen. Palladium has been identified as the most active metal for the HDC reaction of chlorobenzene [8,11,12,13]. However, palladium presents a major problem: the palladium-based catalyst is deactivated by adsorption of the HCl produced during the HDC reaction [14]. Nickel is the metal most often cited after palladium for the HDC of chlorinated compounds [2,11,15,16]. Nickel is less active than palladium but has the advantage of being less sensitive than the latter to deactivation [17]. Some authors have observed an increase in activity and/or stability, i.e., resistance to deactivation, when nickel and palladium are alloyed with other metals such as iron [5]. It is known that certain bimetallic catalysts exhibit superior performance to monometallic catalysts [18,19,20]. Many authors [12,21,22] demonstrated that the selectivity of non-destructive catalytic reactions is often higher for a bimetallic catalyst than a monometallic catalyst. Additionally, bimetallic catalysts are often more resistant to poisoning than monometallic catalysts [11,14]. Among the supports used, carbon [23,24], alumina [11,14] and silica [10,17] are frequently encountered. Only the latter two allow oxidative regeneration of spent catalysts.

There are several existing methods of synthesizing bimetallic catalysts [12]. Among those, the sol–gel method of cogelation allows the synthesis of the support and the dispersion of the precursors of the active metal sites in the porosity of this support to be combined in a single step to produce Ni-Cu/SiO_2_ [22], Cu/SiO_2_ [25], Pd/SiO_2_ [26,27,28] and Fe/SiO_2_ [28,29,30]. The sol–gel method is a synthetic process carried out at a low temperature and at a low pressure which leads to the formation of engineered and controlled ceramic nanopowders. This approach could be successfully employed to fabricate oxide, non-oxide and composite nanopowders with high purity [31]. Previous studies which characterized catalysts prepared using this method have demonstrated the method’s remarkable properties [25,26,28,32]: very high dispersion of the active metallic phase, formation of nanoalloys, localization of metallic nanoparticles in silica cages allowing sintering of the metal to be avoided while keeping it perfectly accessible, hierarchical porous structure allowing easy distribution of reactants and products and the possible regeneration of the catalyst.

The objective of this work is to evaluate the extent to which the sol–gel method of cogelation for the synthesis of catalysts for the HDC of monochlorobenzene is of interest. The novelty resides in the production of Pd-Ni/SiO_2_ and Pd-Fe/SiO_2_ catalysts in one step by the cogelation process, something that is not reported in the literature. Different Pd-Ni and Pd-Fe bimetallic catalysts, supported on silica and synthesized by cogelation, were studied and their performances were evaluated. Then, the performances of the catalysts were related to their physico-chemical properties. A series of characterizations were carried out, including X-ray diffraction, transmission electron microscopy, measurement of nitrogen adsorption–desorption isotherms and ICP. Bimetallic catalysts Pd-Ni/SiO_2_ and Pd-Fe/SiO_2_ and monometallic catalysts Fe/SiO_2_, Ni/SiO_2_ and Pd/SiO_2_ were be synthesized. For each bimetallic catalyst formulation, a series of catalysts with different metal contents was studied, the Pd content remained constant and the value of the second metal varied. Thus, the influence of the presence of the second metal can be studied.

## 2. Results and Discussion

The amounts of all reagents used for material synthesis are detailed in Table 1.

### 2.1. Synthesis

For all catalysts, no weight loss could be observed during the different steps of synthesis, calcination and reduction, and the wt.% of metal was as expected in each material (Table 1). Therefore, all the reagents reacted as was previously observed with this kind of sol–gel synthesis [26,28].

The gel time for all samples is given in Table 2. The gel time is the time which elapsed between the introduction of the last reagent into the solution and gelation in the oven at 80 °C. Gelling is defined as the moment when the liquid no longer flows when the bottle is tilted at an angle of 45°.

As described in [28,34], EDAS reacts faster than TEOS for the hydrolysis and condensation reactions. The evolution of the gel time observed in Table 2 follows this trend. Indeed, for example, in the Pd-Fe series the gel time increases from 22 min to 30 min when the EDAS/TEOS ratio decreases from 0.075 to 0.031. The impact of the EDAS on the catalyst texture will be discussed later when analyzing the specific surface area results (see Section 2.4).

### 2.2. Composition of the Catalysts

**Figure 1:**
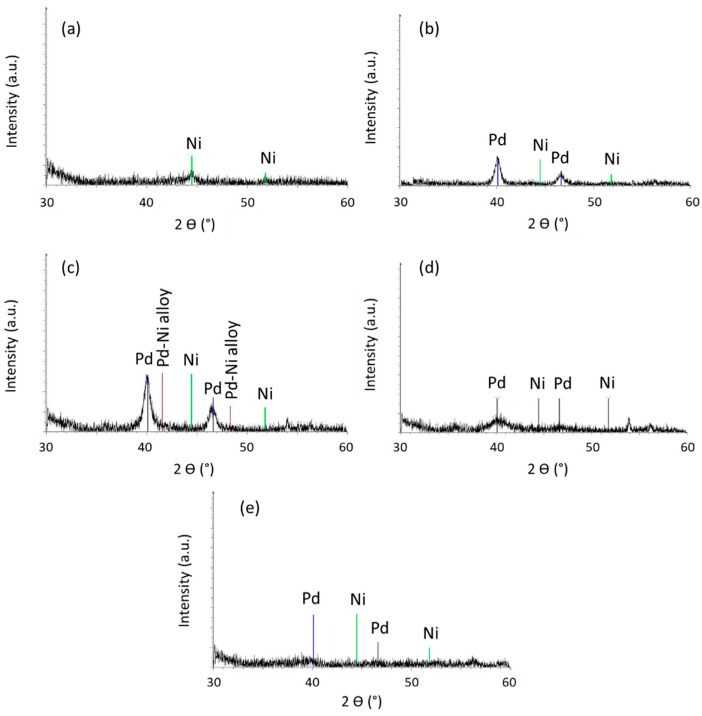
XRD patterns of (**a**) Ni100, (**b**) Pd33Ni67, (**c**) Pd50Ni50, (**d**) Pd67Ni33 and (**e**) Pd100 samples. Reference peaks corresponding to Pd (JCPD No. 46-1043 [24]), Ni (JCPD No. 04-0850 [16]) and Pd-Ni alloy (JCPD No. 65-9444 [35]) are denoted on the figures.

**Figure 2:**
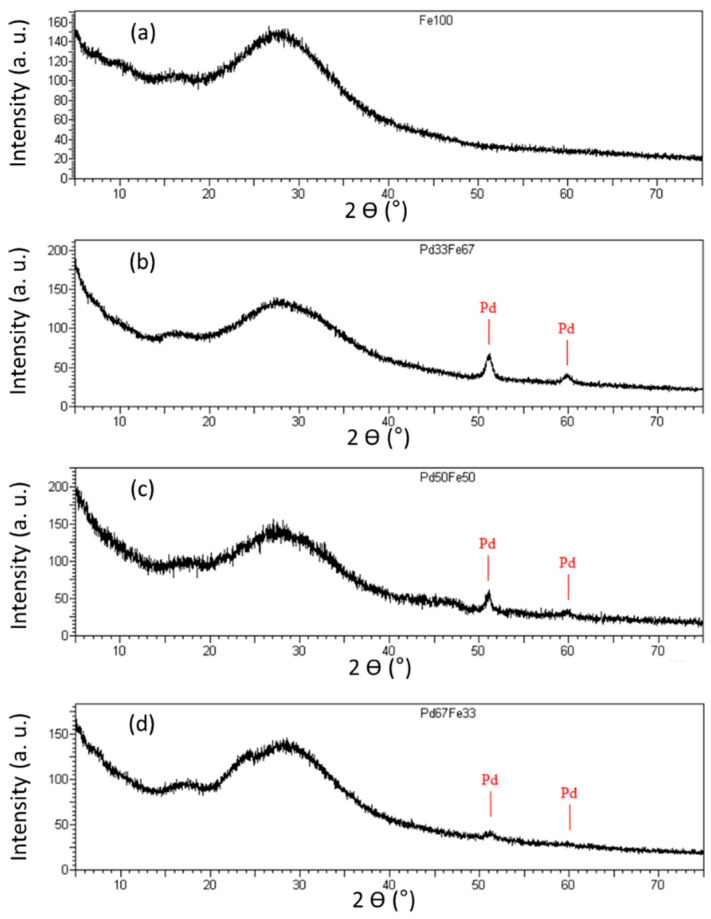
XRD patterns of (**a**) Fe100, (**b**) Pd33Fe67, (**c**) Pd50Fe50 and (**d**) Pd67Fe33 samples. Reference peaks corresponding to Pd are denoted on the figures.

The ICP results (Table 1) showed that the catalysts were well doped with Pd, Ni and Fe but it does not give information about the nature of these species. XRD measurements were performed to determine the phases of the metallic species. Figure 1 and Figure 2 represent all the XRD patterns of the samples.

In Figure 1, the Pd-Ni/SiO_2_ series is represented. In Figure 1a, which represents the Ni100 catalyst, two weak crystallographic reflections are observed coinciding with two of the characteristic lines of Ni at 44.41° and 51.90°. The low intensities of these peaks and their spreading could be explained by two factors: (i) the very small size of the metallic particles; (ii) the presence of amorphous Ni oxide or hydroxide which cannot be detected. In Figure 1b–d which illustrates the Pd-Ni/SiO_2_ catalysts, two crystallographic reflections characteristic of Pd are observed, but none of Ni. The presence of a Pd-Ni alloy would be marked by the presence of a crystallographic reflection between the characteristic lines of Pd and Ni. Their absence at the location of the characteristic peaks of the Pd-Ni alloy and Ni does not mean these two compounds are absent. Indeed, seeing the crystallographic reflection obtained in Figure 1a for the Ni100 sample, one can easily conceive that, with such a weak signal, it is very difficult to detect this compound. Moreover, assuming that a Pd-Ni alloy has been formed, the signal would be even weaker and would more than likely be confused with the background noise.

As regards the Pd-Fe/SiO_2_ series of catalysts, the results obtained are almost identical to the Pd-Ni/SiO_2_ series (Figure 2), except that no compound is detected in the case of the Fe100 sample. As a reminder, the diffractograms in this series extend from 2θ = 0° to 80°. The crystallographic reflections present for angles of less than approximately 40° are due to the support, namely, the silica. The diffractogram corresponding to Fe100 is shown in Figure 2a.

Although a signal can be detected in the case of the Ni100 sample (Figure 1a), no signal is detected in the case of the Fe100 sample (Figure 2a). Two possibilities could explain this phenomenon: (i) the size of the iron particles is very small; (ii) the presence of amorphous iron oxide or hydroxide which cannot be detected. If, with Ni it could already be conceived that the presence of an alloy was possible, with Fe it can be conceived even more easily that a Pd-Fe alloy could be present. Indeed, since Fe was not even detected on the diffractogram for the Fe100 sample (Figure 2a), it is more than likely that if a Pd-Fe alloy existed in the catalysts synthesized for this study, it would not be detected either. Moreover, it seems obvious that if the Fe is not detected in the Fe100 catalyst, it will also not be detected on the other catalysts in the Pd-Fe series, as can be seen on the diffractograms (Figure 2b–d).

The size of the metallic particles (d_XRD_) can be estimated directly from the diffractograms of the catalysts by applying Scherrer’s formula based on the width of the peaks (Equation (3)). The particle sizes of the different catalysts are listed in Table 2 for all the samples. The size is in the range of 5–15 nm for the Pd-Ni/SiO_2_ series and around 20–25 nm for the Pd-Fe/SiO_2_ series.

### 2.3. Morphology

**Figure 3:**
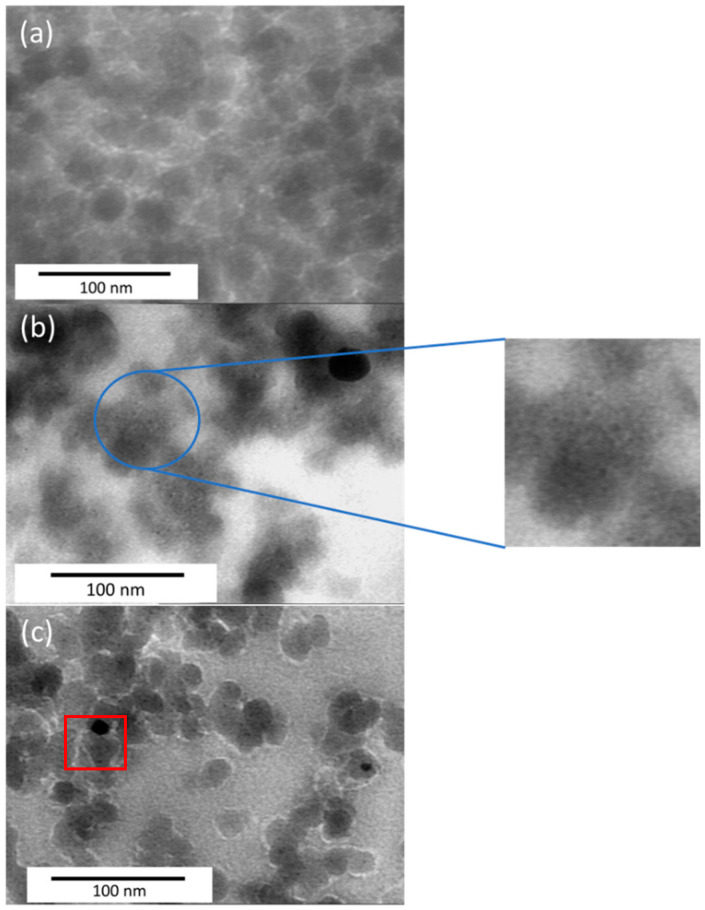
TEM micrographs of (**a**) Ni100, (**b**) Pd33Ni67 and (**c**) Pd50Fe50 samples.

Micrographs of some Pd-Ni/SiO_2_ and Pd-Fe/SiO_2_ catalysts are presented in Figure 3. For most of the samples, similar morphology is obtained with bigger particles (corresponding to silica particles around 20–30 nm) with smaller darker particles inside (metallic particles around 1–3 nm). With the TEM micrographs, one can measure the distribution of the metal particles within the silica matrix and obtain their average metallic size (d_TEM_), as denoted in Table 2. In these images, the metallic particles are generally fairly well distinguishable (zoom on Figure 3b). By measuring the different particles, a mean particle size can be calculated, d_TEM_. For Ni100 (Figure 3a) and Fe100 samples, no metallic nanoparticle is observed inside the silica particle. The presence of oxide in these catalysts, despite the reduction, cannot be ruled out in view of the results obtained using X-ray diffraction. As the contrast is much worse on the TEM images for the oxides, it can be deduced that it would not be possible to distinguish the oxides, which could be one of the explanations for the lack of visible particles for these catalysts. For the other samples, the metallic particle sizes are between 1.5 and 2.6 nm.

It is observed that the average sizes obtained by this method are very different from the sizes determined by X-ray diffraction (Table 2). This can be explained by the presence of very large particles which are relatively rare on the images as can be seen in Figure 3c (red square).

### 2.4. Texture

**Figure 4:**
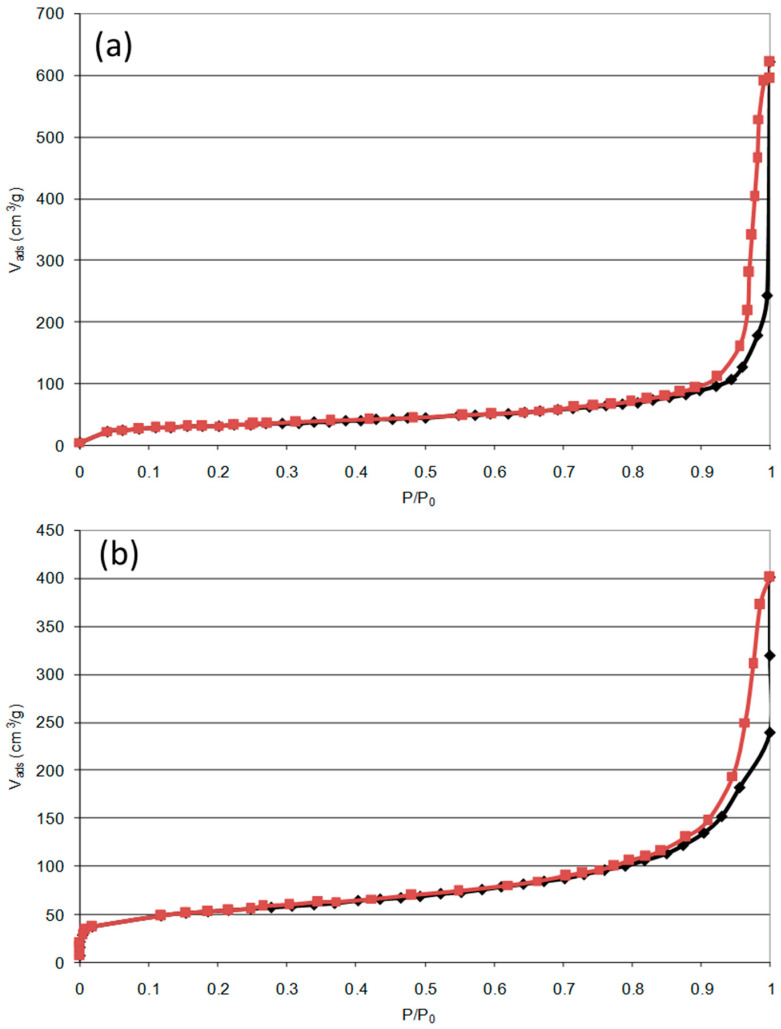
Nitrogen adsorption–desorption isotherms for (**a**) Fe100 and (**b**) Pd50Ni50 samples.

The specific surface area for each sample has been calculated and is denoted in Table 2. Two nitrogen adsorption–desorption isotherms are represented in Figure 4 for the Fe100 and Pd50Ni50 samples, illustrating the two shapes observed for all samples. Indeed, for all samples except for the Fe100 catalyst, the isotherms look like Figure 4b with a sudden increase in adsorbed volume at low relative pressures followed by a plateau, characteristic of microporous adsorbents (type I [36]). In the case of the Fe100 sample (Figure 4a), at low relative pressures no sudden increase in absorbed volume is observed, which testifies to the absence of micropores. At relative pressures close to 1, the isotherms correspond in all cases to a type IV isotherm (macroporous adsorbents [36]).

All the isotherms present a hysteresis characteristic of the phenomenon of capillary condensation in the mesopores.

For the S_BET_ values, it can be observed that in each series the specific surface area increases with the EDAS/TEOS ratio as previously explained in [26,28,29,34]. This is due to the role of the nucleating agent EDAS which reacts faster than TEOS (see gel time in Table 2 and Section 2.1) and is therefore the nucleating agent of the silica particles. As was previously described in [26,28,29,34], when the amount of EDAS increases it leads to a higher specific surface area. For example, in the Pd-Fe/SiO_2_ series (Table 2), when the EDAS/TEOS ratio is equal to 0.031 for the Pd67Fe33 sample, the S_BET_ value is 300 m^2^/g, while when the EDAS/TEOS ratio increases to 0.075 for the Pd33Fe67 sample, the S_BET_ is 395 m^2^/g.

### 2.5. Catalytic Activity

It should be noted that the benzene selectivity is equal to 100% for each catalyst evaluated in this study.

#### 2.5.1. Pd-Ni/SiO_2_ Series

**Figure 5:**
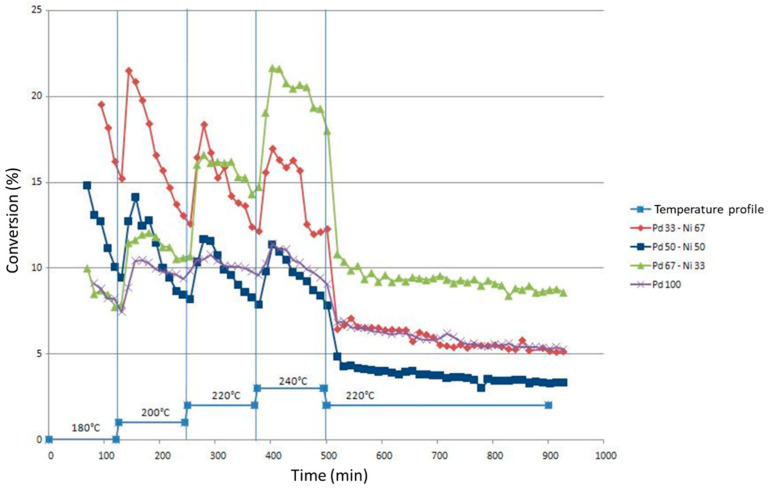
Comparison of the conversions of chlorobenzene to benzene as a function of time for the Pd-Ni/SiO_2_ series.

Figure 5 shows the conversion of chlorobenzene to benzene as a function of time for the different catalysts in the Pd-Ni/SiO_2_ series. The mean conversion values on the plateau at 220 °C are denoted in Table 3 for all samples.

It must be remembered that the mass of the catalyst for the Pd-Ni/SiO_2_ series is 0.2 g compared to the Pd-Fe series and the Pd monometallic sample where it is 0.1 g. This difference in mass is necessary for the Pd-Ni/SiO_2_ series as otherwise the initial conversion is too high and leads to a rapid deactivation.

There are two different types of behavior in this same series: (i) a relatively constant activity on each level, that is to say a fairly low deactivation. This behavior is characteristic for Pd100 and Pd67Ni33 catalysts; (ii) a strong deactivation. The increase in temperature increases the activity of the catalyst. The deactivation is such that this increase does not make it possible to obtain a better conversion than the maximum conversion at the previous level. This behavior is characteristic of the Pd33Ni67 and Pd50Ni50 catalysts.

Note that the Ni100 catalyst is not shown in Figure 5. In fact, the catalytic experiment carried out on this catalyst revealed virtually zero activity throughout the test, despite the temperature variations (less than 1%).

Taking into account the mass difference introduced into the reactor between the Pd-Ni/SiO_2_ series and the monometallic Pd catalyst, it is observed that the presence of Ni in the catalyst does not improve the conversion of chlorobenzene into benzene. Indeed, given that in the case of the Pd100 sample a mass of catalyst twice as small as the mass introduced into the reactor for the Pd-Ni/SiO_2_ series samples was introduced into the reactor, one should obtain a significantly lower conversion of chlorobenzene to benzene than in the case of bimetallic catalysts. However, in Figure 5, it is observed that the conversion values of chlorobenzene into benzene for the Pd100 sample are similar to those obtained for the Pd50Ni50 sample, whereas the mass present in the reactor for the Pd100 catalyst is double that for the Pd50Ni50 sample.

When the nickel content increases in the bimetallic catalysts, the deactivation of the bimetallic catalyst takes place more and more rapidly. Therefore, the Pd67Ni33 catalyst is the most attractive of the Pd-Ni/SiO_2_ catalysts tested. Seeing the increase in conversion with temperature, it could be thought that this catalyst would only become more interesting than the monometallic Pd catalyst at higher temperatures.

The differences in activity and behavior between the different Pd-Ni/SiO_2_ catalysts could be the consequence of the formation of different alloys, the nature of which differs according to the quantity of Ni present in the bimetallic catalyst.

**Figure 6:**
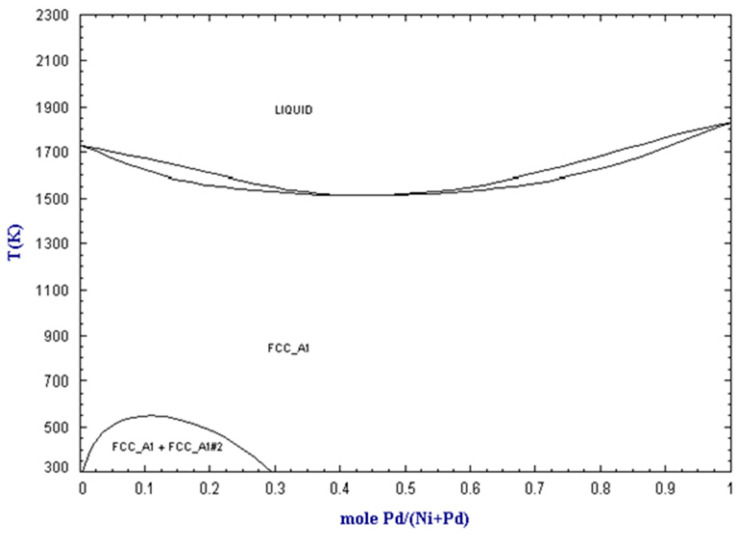
Phase diagram of the Pd-Ni alloy, reproduced from [38].

According to the phase diagram of the Pd-Ni alloy (Figure 6), at the temperatures of the hydrodechlorination reaction of chlorobenzene to benzene (between 453 K and 513 K), Pd and Ni form a solid solution, and this is the case in the entire range of concentration between pure Pd and pure Ni. According to this phase diagram, the Pd-Ni/SiO_2_ catalysts studied in this work therefore form an alloy with an FCC structure (face centered cubic). However, since the metal particles present in the Pd-Ni/SiO_2_ catalysts are very small in size (d_TEM_, Table 2), the metals may not follow exactly the same phase diagram [37]. Nevertheless, in Figure 1, the main palladium lines appear. According to Scherrer’s formula as applied to these peaks (Equation (3)), particle sizes between 4 and 13 nm are obtained (d_XRD_, Table 2). It could therefore be that the large particles observed using TEM (Figure 3c) are particles of pure palladium. It is also hypothesized that the small particles which are not detectable by using X-ray diffraction consist of a mixture of the two metals, Pd and Ni.

The Pd-Ni alloy is an alloy that has been studied very little in the literature on the hydrodechlorination reaction of chlorinated compounds. These catalysts are reported, as is the case with this study, to provide good activity, which, however, is lower than in the case of Pd-Fe catalysts. However, Pd-Ni catalysts have a selectivity close to or equal to 100% for the hydrodechlorination of chlorinated compounds [12,39].

An explanation for the better activity of catalysts containing a large amount of Ni has been given by Simagina et al. [39]. The cause may be a Pd segregation phenomenon on the surface of the bimetallic particles. At a high Ni concentration, the Pd is essentially found isolated in small aggregates on the surface of the Ni-rich particles and is therefore more active than in a case where it is found in larger aggregates. Indeed, according to Śrębowata et al. [40], there would be a segregation of Pd at the surface of the Pd-Ni particles.

The deactivation of catalysts containing Ni has already been observed in previous works [2,41]. The cause of this deactivation seems to be the adsorption of HCl. The adsorbed HCl modifies the cubic crystalline structure of Ni to form a hexagonal structure of the NiCl_2_ type which induces an irreversible deactivation of the catalyst. The deactivation of Pd-based catalysts supported on alumina is usually caused by coking, which leads to a rapid decrease in catalytic activity [12,42]. Nevertheless, in this work it can be assumed that coking is a minor phenomenon on the silica support [12,19] and that catalyst deactivation is mainly due to Ni deactivation by chlorine atoms. This would explain the increased deactivation of the catalysts when the Ni content increases.

#### 2.5.2. Pd-Fe/SiO_2_ Series

**Figure 7:**
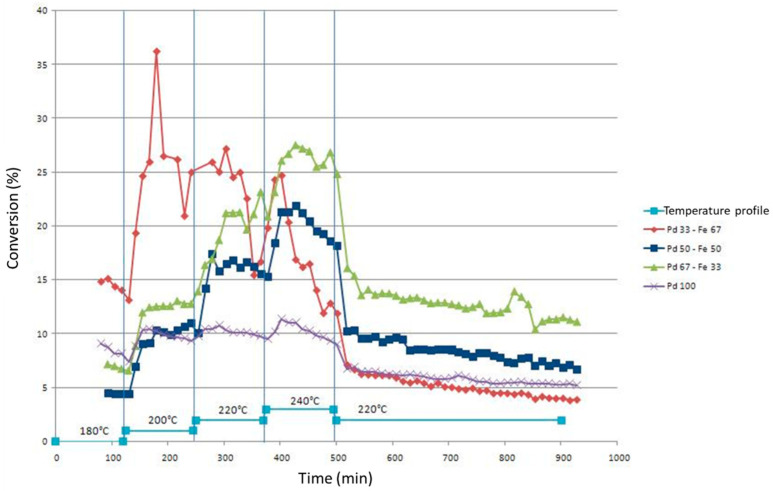
Comparison of the conversions of chlorobenzene to benzene as a function of time for the Pd-Fe/SiO_2_ series.

Figure 7 shows the conversion of chlorobenzene to benzene for the different catalysts tested in the Pd-Fe/SiO_2_ series. The mean conversion values on the plateau at 220 °C are denoted in Table 3 for all samples.

Again, the same two types of behavior observed for the Pd-Ni/SiO_2_ series are present, namely: (i) a relatively constant activity on each level, i.e., a fairly low deactivation. This behavior is characteristic of Pd100, Pd67Fe33 and Pd50Fe50 catalysts; (ii) a strong deactivation. The increase in temperature increases the activity of the catalyst. The deactivation is such that this increase does not make it possible to obtain a better conversion than the maximum conversion at the previous level. This behavior is characteristic of the Pd33Fe67 catalyst, other than between the first two levels at 180 °C and 200 °C.

It can be noted that the Fe100 catalyst is not shown in Figure 7. Indeed, the catalytic experiment carried out on this catalyst revealed zero activity throughout the test despite the temperature variations.

In Figure 7, it is observed that the presence of Fe in the presence of Pd leads to different results depending on the quantity of Fe present in the bimetallic catalyst: (i) When compared to the Pd100 catalyst, the presence of a small amount of iron (Pd67Fe33) improves the performance of the catalyst without affecting its deactivation; (ii) the presence of a quantity of Fe equivalent to the quantity of Pd in the catalyst Pd50-Fe50 reduces catalytic performance at a low temperature (180 °C and 200 °C). The conversion values of chlorobenzene into benzene are only higher than those of the Pd100 sample from the third stage, namely 220 °C. Deactivation remains low; (iii) the presence of a greater quantity of Fe than of Pd (Pd33Fe67) improves catalytic performance, compared to the Pd100 and Pd67Fe33 catalysts. On the other hand, the deactivation of the catalyst is much stronger than in the other Pd-Fe/SiO_2_ catalysts.

According to the literature [43], the difference in the behavior of a catalyst with the presence of a second metal can be explained by the formation of alloys. The change in the electronic state of the catalyst would then be responsible for the modification of the catalytic properties [44].

**Figure 8:**
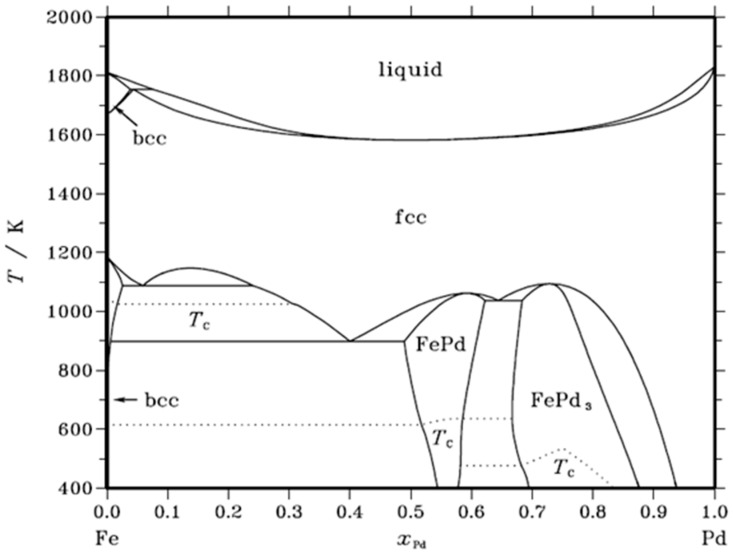
Phase diagram of the Pd-Fe alloy, reproduced from [38].

On the Fe-Pd phase diagram [38] (Figure 8), by tracing a horizontal line at 400 °C (673 K), there are several possible alloys depending on the atomic percentage of Pd: a mixture of Fe + FePd, the FePd alloy or the FePd_3_ alloy. It is hypothesized that the nature of the particles detected on micrographs (Figure 3) follows this phase diagram, although the metallic particles are very small in size [37].

Pure palladium particles were detected by using XRD (Figure 2). According to Scherrer’s formula (Equation (3)), the dimensions of these pure palladium particles are between 22 and 25 nm (Table 2). These particles would thus correspond to the large particles observed by TEM. As these are quite rare (Figure 3c), it is considered that the hydrodechlorination activity of Pd-Fe/SiO_2_ catalysts mainly result from the small metal particles (Table 2).

For a percentage close to 33 atomic % in Pd, which would correspond to the catalyst Pd33Fe67, the palladium and the iron are organized in a Fe + FePd mixture. The same analysis can be carried out for the two other catalysts, Pd50Fe50 and Pd67Fe33, therefore corresponding, respectively, to 50% and 67% atomic Pd. In these two cases, the alloys present are in different proportions or in different natures. For the Pd50-Fe50 sample, the FePd alloy would be almost alone with a small proportion of monometallic Fe, while for the Pd67Fe33 sample a mixture of alloys with FePd and FePd_3_ structure is present.

In view of these results (Figure 7), it seems that the FePd-type alloy only allows a better conversion of chlorobenzene into benzene at higher temperatures than for the Pd100 sample. The presence of iron in the unalloyed state, although not active if used alone in a catalyst, could greatly favor the conversion of chlorobenzene into benzene but nevertheless leads to accelerated deactivation of the catalyst. Finally, the FePd_3_ structural alloy shows better activity than the FePd structural alloy while keeping a reasonable deactivation rate.

Zhang et al. [45] suggest an explanation for the roles played by the two metals. The Pd would act as a catalyst while the Fe would act as an electron donor (reducer). According to Kim et al. [46], the presence of Fe improves the activity of Pd-based catalysts. Tests revealed an alloy with an FePd structure. This alloy would be responsible for the improvement of the catalytic activity because of the cooperative effect of Fe during the hydrogen transfer step.

According to some authors [9,47,48], the particles of Pd-Fe catalysts would be enriched at the surface with Fe atoms and therefore the formation of FeCl_3_ on the surface of the catalysts would be thermodynamically favored compared to the formation of PdCl_2_. In this case, the poisoning of Pd sites by HCl is reduced by the elimination of chlorine in the form of FeCl_3_. In this way, the stability of the bimetallic catalysts is higher than that of the monometallic catalyst based on Pd, as is the case here for the catalysts Pd67Fe33 and Pd50Fe50. Too high a quantity of Fe can nevertheless lead to a phenomenon of encapsulation of the Pd active sites by FeCl_3_. This would lead to lower stability than in the case of Pd alone, as is the case with Pd33Fe67 [43].

Lingaiah et al. [47] also highlight the importance of particle sizes in the context of the HDC of chlorobenzene. Low dispersion would improve the activity of the catalysts tested, thus demonstrating that the hydrodechlorination reaction of chlorobenzene is sensitive to the surface structure of the catalysts. In addition, interest in low dispersion would be based on an improvement in catalytic stability. Large Pd particles would be less prone to quenching by HCl. These results show that the process of drying using microwaves (MW) is of particular interest. This technique would favor the increase of particle sizes compared to traditional thermal drying methods. In the case of this present study, the metal particles remain small in size, and the activity remains high.

Lingaiah et al. [47] have also shown that even catalysts with high dispersion can exhibit activity and stability comparable to catalysts with low dispersion. Their findings also show a higher activity of monometallic Pd catalysts compared to those of bimetallic catalysts. However, Babu et al. [5] highlight the possibility of forming particular Pd species which are more active than others. It could therefore be thought that the species formed in this work are much more active in the case of bimetallic alloys than for monometallic Pd.

### 2.6. Comparison with the Literature

In Table 4, the present study is compared to the literature by summarizing the most important parameters and results of each study. First, it can be observed that it is quite difficult to make a comparison because many conditions such as the temperature, the amount of the catalyst, or the H_2_ fraction differ greatly from one study to another. From Table 4, it can be also seen that some studies do not give all the information needed for a proper comparison.

Second, it is observed that the catalysts developed in this paper allow a 100% selectivity at a reasonably low temperature (220 °C) with a dilute amount of H_2_ (2.6 vol%). Conversion is lower, but the idea of this study was to stay at lower conversion in order to consider a differential reactor. The benzene selectivity is often missing in the other studies and, as expected, conversion increases when the temperature is higher.

## 3. Conclusions

Two series of bimetallic catalysts, Pd-Ni/SiO_2_ and Pd-Fe/SiO_2,_ were synthesized by the sol–gel process for the catalysis of the hydrodechlorination of chlorobenzene into benzene. This synthesis process uses two silicon alkoxides, TEOS and EDAS. EDAS is a modified alkoxide with a diamine function allowing the metal to be highly dispersed in the silica matrix. In all cases, the sol–gel process allows very small metallic nanoparticles in the order of 2–3 nm to be dispersed inside the silica matrix. Nevertheless, the presence of some large particles of pure Pd in the analyzed catalysts can be noted. The catalysts have a specific surface between 100 and 400 m^2^/g.

All bimetallic catalysts can catalyze the hydrodechlorination of chlorobenzene. In order to consider a differential reactor, the work was carried out at low conversion conditions. The Pd-Ni combination is nevertheless less active than the Pd-Fe combination. Monometallic catalysts Ni/SiO_2_ and Fe/SiO_2_ are inactive for the hydrodechlorination reaction of chlorobenzene, with no conversion.

Pd-Ni/SiO_2_ catalysts are less active than monometallic Pd catalyst (mean conversion of 6%). For catalysts with a low proportion of Ni such as Pd67Ni33 (mean conversion of 9%), one can only foresee a possible improvement in activity compared to that of the monometallic Pd catalyst for reaction temperatures above 240 °C. In this series of catalysts, increasing the Ni content increases the activity but leads to an amplification of the catalyst deactivation phenomenon compared to Pd alone (mean conversion drops to 4% with the Pd50Ni50 sample). The increase in activity could be due to an overall effect, i.e., the Ni would dilute the Pd into small aggregates, and thus increase the surface area of active Pd. The deactivation would be due to the formation of NiCl_2_.

The Pd-Fe catalysts are overall more active than the monometallic Pd catalyst. The difference in the results obtained for each of the catalysts in the series could be explained by the significant presence of FePd alloy in the catalyst. The combined action of Fe and Pd would be responsible for this increase in activity. Fe would have a cooperative effect when associated with Pd, although it is inactive alone. The presence of Fe could also reduce the phenomenon of Pd poisoning by HCl. The best Pd-Fe catalyst (Pd67Fe33) doubled the mean conversion to 13% compared to the Pd monometallic catalyst (mean conversion of 6%).

## 4. Materials and Methods

### 4.1. Catalyst Synthesis

Reagents: Fe(III) acetylacetonate (Fe(acac)_3_, (CH_3_COCH=C(O)CH_3_)_3_Fe, Sigma-Aldrich, 97%, St. Louis, MO, USA); Pd (II) acetylacetonate (Pd(acac)_2_, CH3COCH=C(O)CH_3_)_2_Pd, Sigma-Aldrich, 99%); Ni(II) acetylacetonate (Ni(acac)_2_, CH_3_COCH=C(O)CH_3_)_2_Ni, Sigma-Aldrich, 97%); Ammonia solution (NH_4_OH, Sigma-Aldrich, 25%); distilled water; tetraethoxysilane (TEOS, Si(OC_2_H_5_)_4_, Sigma-Aldrich, 98%); N-[3-(triméthoxysilyl)propyl]ethylenediamine (EDAS, (OCH_3_)_3_-Si-(CH_2_)_3_-NH-(CH_2_)_2_-NH, Sigma-Aldrich, 97%). The amounts of all reagents used for material synthesis are detailed in Table 1.

Pd(acac)_2_ and the salt of the second metal, (Ni(acac)_2_ or Fe(acac)_3_), were mixed with EDAS and half the volume of ethanol (or only one metallic salt if a monometallic catalyst was prepared). The mixture was then stirred at room temperature until the solution became clear. After the addition of TEOS, a 0.54 M aqueous ammoniacal solution diluted in the remaining half of the ethanol was added with vigorous stirring. The synthesis bottle was then hermetically sealed and placed in the oven at 80 °C for 3 days in order to undergo gelling and aging of the gel.

**Figure 9:**
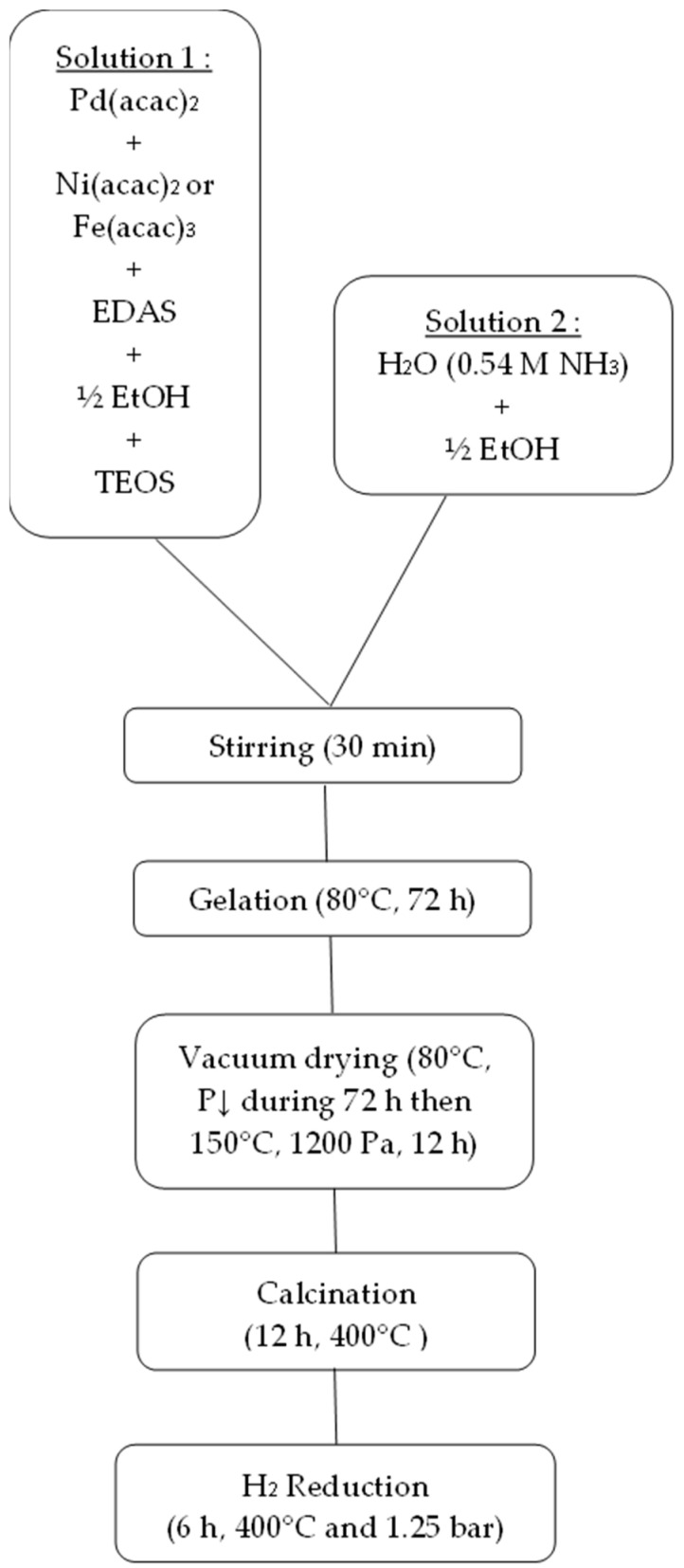
Schematic diagram of the catalyst preparation.

The wet gels were then dried under vacuum at 80 °C for 3 days, during which the pressure was slowly reduced to 1200 Pa in order to avoid the gel exploding. The gels were then dried at 150 °C at 1200 Pa for 12 h. The dried gels were then calcined in air (0.1 mmol/s) for 12 h at 400 °C with a ramp of 120 °C/h (air flow rate 0.02 mmol/s). The calcined gels used for the catalytic tests were finally reduced in situ under hydrogen (0.05 mmol/s) for 6 h at 400 °C and 1.25 bar. The catalysts used for the characterization were reduced under hydrogen (0.25 mmol/s) for 6 h at 400 °C and at atmospheric pressure. The synthesis process is represented in Figure 9.

### 4.2. Characterizations

Nitrogen adsorption–desorption isotherms were obtained thanks to an ASAP 2420 multi-sampler adsorption–desorption volumetric device from Micromeritics.

The crystallographic properties were observed through the X-ray diffraction (XRD) patterns recorded with a Siemens D5000 powder diffractometer using Cu-K_α_ radiation for the Ni series and using Fe-K_α_ radiation for the Fe series. The Scherrer formula (Equation (3)) was used to determine the size of the metal crystallites (i.e., *d*_XRD_) [33]:

(3) $$d_{XRD}=0.9\frac{\lambda }{\left(B\cos(\theta )\right)}$$

where *d*_XRD_ is the crystallite size of metals (nm), *B* the peak full width at half maximum after correction of the instrumental broadening (rad), *λ* the wavelength (nm) and *θ* the Bragg angle (rad).

The sizes of the metallic nanoparticles were estimated by transmission electron microscopy (TEM) with a Philips CM100 device, with measurements being taken on approximately one hundred particles. Each sample was prepared before being analyzed using TEM. This preparation was necessary to disperse the samples and thus allow their analysis under the electron microscope. The preparation procedure was as follows:

(i) the catalysts to be observed were embedded in the EPON 812 resin;
(ii) embedded catalysts were placed under vacuum for a few hours to allow the resin to penetrate the pores of the catalyst;
(iii) the catalysts then remained for 3 days at 60 °C for the resin to polymerize;
(iv) very thin slices of polymerized resin containing the catalyst were cut using a crystal knife;
(v) this thin slice was then placed on a copper grid.

The grids had a diameter of 3.05 mm and included 400 mesh (37 µm thick).

The sections were then placed directly on the sample holder of the transmission electron microscope.

Real palladium, nickel and iron contents in the catalysts were measured using inductively coupled plasma atomic emission spectroscopy (ICP-AES) on a Spectroflam from Spectro Analytical Instruments. Samples were dissolved in concentrated acids (18 M H_2_SO_4_, 22 M HF, 14 M HNO_3_). Palladium, nickel and iron contents were obtained by comparison with standard solutions in the same medium.

### 4.3. Catalytic Experiments

**Figure 10:**
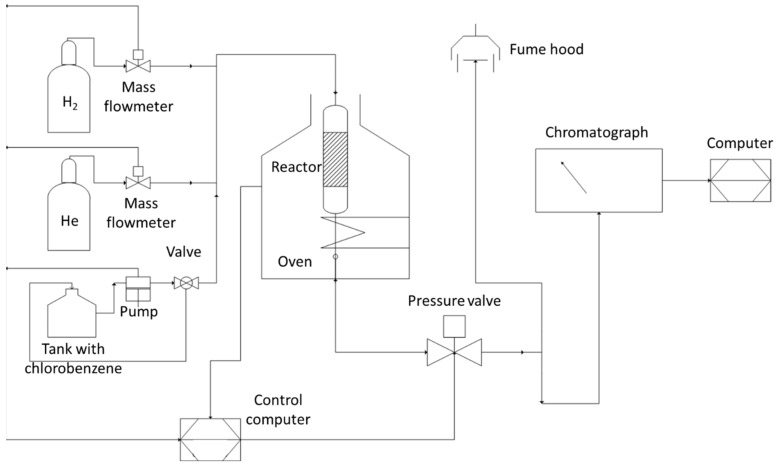
Installation Scheme.

The installation used for the hydrodechlorination of chlorobenzene is represented in Figure 10. The reactor was removed and charged with 0.2 g of Pd-Ni/SiO_2_ catalyst or 0.1 g of Pd-Fe/SiO_2_ catalyst. It was then moved back to its original location. The system was purged with helium. After a purge time of 5 to 10 min, the reduction procedure could be started: a temperature ramp of 5 °C/min up to a plateau of 400 °C was maintained for 6 h, the 2 stages being carried out under a flow rate of 4 *N*L/h of hydrogen. The reduction temperature was determined by temperature programmed reduction (TPR) (see Supplementary Materials). The temperature of the oven was then brought back to 180 °C, the starting temperature of the actual test cycle. The hydrogen flow was reduced to 1 *N*L/h and a flow of 37 *N*L/h of helium was added.

**Figure 11:**
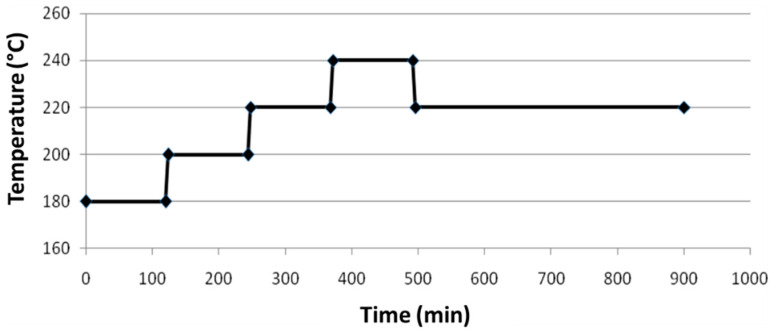
Temperature profile used for the catalytic experiments.

The temperature program was made up of different levels of 180 °C, 200 °C, 220 °C, 240 °C and finally a return to 220 °C. Each level was maintained for a period of 2 h except for the last which was maintained for a minimum of 6 h. The temperature ramps were set at a rate of 5 °C/min. An illustration of this temperature program can be seen in Figure 11. The start of the program coincided with the pivoting of the valve, allowing continuous injection of chlorobenzene.

Throughout the experiment, the chromatograph analyzed the reactor effluents at regular intervals, i.e., approximately every 12 min. Each experiment was carried out three times to assess reproducibility. The effluent was analyzed by gas chromatography (ThermoFinnigan with FID) using a Porapak Q5 packed column.

## Figures and Tables

**Table 1:** Operating synthesis variables for the catalysts Pd-Fe/SiO_2_ et Pd-Ni/SiO_2_.

	**Pd Amount (wt.%) ^a^**	**Fe Amount (wt.%) ^a^**	**n_Pd(NO3)2_** **(mmol)**	**n_Fe(acac)3_** **(mmol)**	**n_TEOS_** **(mmol)**	**n_EDAS_** **(mmol)**	**n_water+NH3_** **(mmol)**	**n_C2H5OH_** **(mmol)**
Pd100	1.50	-	0.96014	-	109.71	1.92	555.76	1116.31
Pd33-Fe67	1.50	1.57	0.97731	1.95463	103.99	7.82	549.28	1118.11
Pd50-Fe50	1.50	0.79	0.96865	0.96865	106.88	4.84	552.55	1117.21
Pd67-Fe33	1.50	0.39	0.96438	0.48219	108.30	3.37	554.16	1116.76
Fe100	-	0.79	-	0.95349	108.80	2.86	554.16	1116.76
	**Pd Amount (wt.%) ^a^**	**Ni Amount (wt.%) ^a^**	**n_Pd(NO3)2_** **(mmol)**	**n_Ni(acac)3_** **(mmol)**	**n_TEOS_** **(mmol)**	**n_EDAS_** **(mmol)**	**n_water+NH3_** **(mmol)**	**n_C2H5OH_** **(mmol)**
Pd33-Ni67	1.50	1.66	2.44925	4.89851	245.69	34.29	357.03	2799.78
Pd50-Ni50	1.50	0.83	0.96982	0.96982	104.05	7.76	549.35	1118.10
Pd67-Ni33	1.50	0.41	2.41239	1.2062	267.24	12.06	381.42	2793.00
Ni100	1.50	0.83	-	0.995462	106.02	5.73	551.58	1117.48

^a^ = measured by ICP-AES.

**Table 2:** Physico-chemical properties of samples Pd-Fe/SiO_2_ and Pd-Ni/SiO_2_.

	**Gel Time** **(min)**	**d_XRD_** **(nm) ^a^**	**d_TEM_** **(nm)**	**S_BET_** **(m^2^/g)**	**[EDAS]/[TEOS]**
Pd100	55	-	2.1	220	0.018
Pd33Fe67	22	25	1.5	395	0.075
Pd50Fe50	25	22	2.1	360	0.045
Pd67Fe33	30	19	2.6	300	0.031
Fe100	35	-	-	110	0.026
	**Gel Time** **(min)**	**d_XRD_** **(nm) ^a^**	**d_TEM_** **(nm)**	**S_BET_** **(m^2^/g)**	**[EDAS]/[TEOS]**
Ni100	30	6	-	230	0.054
Pd33Ni67	18	13	2.6	320	0.140
Pd50Ni50	25	11	2.5	290	0.075
Pd67Ni33	35	4	2.5	250	0.045

^a^ = measured with Scherrer equation [33].

**Table 3:** Mean chlorobenzene conversion at 220 °C.

	**Chlorobenzene Conversion (%)**	**Standard Deviation (%)**
Pd100	6.0	0.7
Pd33Fe67	5.3	1.1
Pd50Fe50	8.3	1.1
Pd67Fe33	12.5	1.0
Fe100	0.0	0.0
	**Chlorobenzene Conversion (%)**	**Standard Deviation (%)**
Ni100	0.0	0.0
Pd33Ni67	6.0	0.7
Pd50Ni50	3.7	0.6
Pd67Ni33	8.7	0.9

**Table 4:** Comparison of the chlorobenzene (CB) catalytic activity with the literature.

References	Catalyst (Optimal Active Phase Content)	Catalytic Experiment Conditions	Optimal Catalytic Results
Present study	Pd-Ni/SiO_2_ (1.5 wt% of Pd and 0.41 wt% of Ni) Pd-Fe/SiO_2_ (1.5 wt% of Pd and 0.39 wt% of Fe)	Temperature 220 °C Catalytic mass of 0.1 g (for Fe series) or 0.2 g (for the Ni series) vol% of H_2_ with H_2_:CB molar ratio of 1000:1	12.5% conversion at 220 °C with a benzene selectivity of 100% for the best of Ni series 9% conversion at 220 °C with a benzene selectivity of 100% for the best of Fe series
[2]	Ni/Al_2_O_3_ (6 wt% of Ni)	Temperature range 150–350 °C Catalytic mass of 0.1 g H_2_:CB molar ratio of 50:1	100% conversion at 300 °C with a benzene selectivity of 20%
[16]	Ni/Al_2_O_3_ (90 wt% of Ni)	Temperature range 350–650 °C Catalytic mass of 0.1 g 97 vol% of H_2_	100% conversion at 350 °C with a benzene selectivity of 100% under pure H2 flow
[5]	Pd-Fe/Al_2_O_3_ (0.5 wt% of both Pd and Fe)	Temperature 140 °C Catalytic mass of 1 g H_2_:CB molar ratio of 10:1	Conversion not specified Selectivity of benzene of 98%
[9]	Pd-Fe/Carbon (2 wt% of both Pd and Fe)	Temperature range 150–200 °C Catalytic mass not specified H_2_:CB molar ratio of 3:1	100% conversion at 200 °C Selectivity not specified
[10]	Ni/MCM-41 (10 wt% of Ni)	Temperature 100 °C Catalytic mass not specified H_2_ partial pressure of 1.5 MPa	100% conversion at 200 °C Selectivity not specified
[11]	Pd-Ni/Al_2_O_3_ (0.005 wt% of both Pd and Ni)	Temperature range 150–350 °C Catalytic mass of 50 mg H_2_:CB molar ratio of 50:1	87% conversion at 200 °C Selectivity not specified
[14]	Pd-Ni/Al_2_O_3_ (0.5 wt% of both Pd and Ni)	Temperature 140 °C Catalytic mass of 0.8 g H_2_:CB molar ratio of 3:1	100% conversion at 140 °C Selectivity not specified
[17]	Ni_2_P/SiO_2_ (5 wt% of Ni)	Temperature range 250–400 °C Catalytic mass of 0.5 g H_2_ flow of 60 mL/min	100% conversion at 325 °C Selectivity not specified
[23]	Pd/Carbon (0.9 wt% of Pd)	Temperature range 100–300 °C Catalytic mass of 8 mg H_2_:CB molar ratio of 30:1	85% conversion at 300 °C Selectivity not specified

## Data Availability

The raw/processed data required to reproduce these findings cannot be shared at this time as these data are part of an ongoing study.

## Supplementary Materials

The following supporting information can be downloaded at: https://www.mdpi.com/article/10.3390/gels9040275/s1, Figure S1. TPR of Pd50-Ni50 sample; Figure S2. TPR of Pd50-Fe50 sample.
