# Supplementary material for: Green Chemistry for the Transformation of Chlorinated Wastes: Catalytic Hydrodechlorination on Pd-Ni and Pd-Fe Bimetallic Catalysts Supported on SiO2
Source: Gels. 2023 Mar 25;9(4):275. doi: 10.3390/gels9040275 (PMC10137916; doi:10.3390/gels9040275)
Supplement: Supplementary file 1 [file gels-09-00275-s001.zip › gels-2291672-supplementary.pdf]

gels

MDPI

Article

Green Chemistry for the Transformation of Chlorinated Wastes: Catalytic Hydrodechlorination on Pd-Ni and Pd-Fe Bimetallic Catalysts Supported on SiO_2

Julien G. Mahy[1,2*], Thierry Delbeuck[1], Kim Yên Tran[1], Benoît Heinrichs[1] and Stéphanie D. Lambert[1]

[1] Department of Chemical Engineering – Nanomaterials, Catalysis & Electrochemistry, University of Liège, B6a, Quartier Agora, Allée du six Août 11, 4000 Liège, Belgium
[2] Institut National de la Recherche Scientifique (INRS), Centre-Eau Terre Environnement, Université du Québec, 490, Rue de la Couronne, Québec, QC G1K 9A9, Canada
* Correspondence: julien.mahy@uliege.be; Tel.: +32-3663563

Supplementary Materials of

Determination of the reduction procedure of the samples by temperature programmed reduction (TPR)

1. Methods

Temperature programmed reduction (TPR) analysis can determine the number of reducible species present in a sample and the temperature at which reduction takes place as a function of gas flow rate, percentage of reactive gas, amount of sample and the rate of temperature increase. The TPR analyzes are carried out on a CE Instruments TPD/R/O 1100 Xsorb.

The samples analyzed are first calcined at a temperature of 400 °C. These samples are then crushed and placed in a quartz tube, one end of which is made of sintered glass. The amount of sample inserted into the device is equal to 0.2 g.

Before being reduced, the samples are first purged under a nitrogen gas stream for 15 min.

The gas used for TPR analysis is a mixture of reactive gas with an inert gas. In this case, we took a gas whose composition is 5%/95% H_2/N_2. During the test, the samples are subjected to a temperature rate of 10°C/min until they reach 400 °C. The samples stay at this temperature for 8 h. The flow rate of the gas mixture circulating through the sample is constant and fixed at 20 cm^3/min.

At the reduction temperature of present species, hydrogen consumption peaks appear. Hydrogen consumption is monitored by a TCD detector.

2. Results

Following the TPR analysis, the following procedures were chosen for the reduction of Pd-Ni/SiO2 catalysts and Pd-Fe/SiO2 catalysts:

The catalysts used for the catalytic tests are reduced in situ in the hydrodechlorination installation under hydrogen (4 NL/h) for 6 h at 400 °C and 1.25 bar;

The catalysts used for the characterization are reduced in a vertical tubular furnace under hydrogen (20 NL/h) for 6 h at 400 °C. and at atmospheric pressure.

The TPR results for the samples Pd50-Ni50 and Pd50-Fe50 , as example, are shown in Figures S1 and S2.

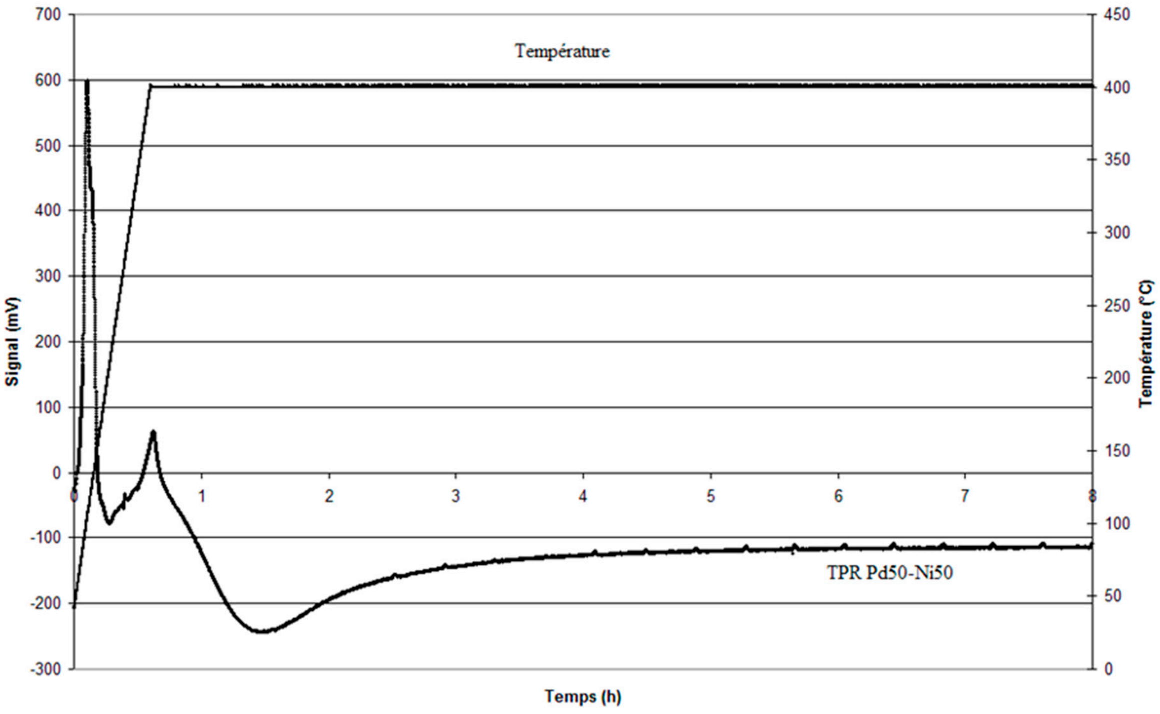

Figure S1. TPR of Pd50-Ni50 sample.

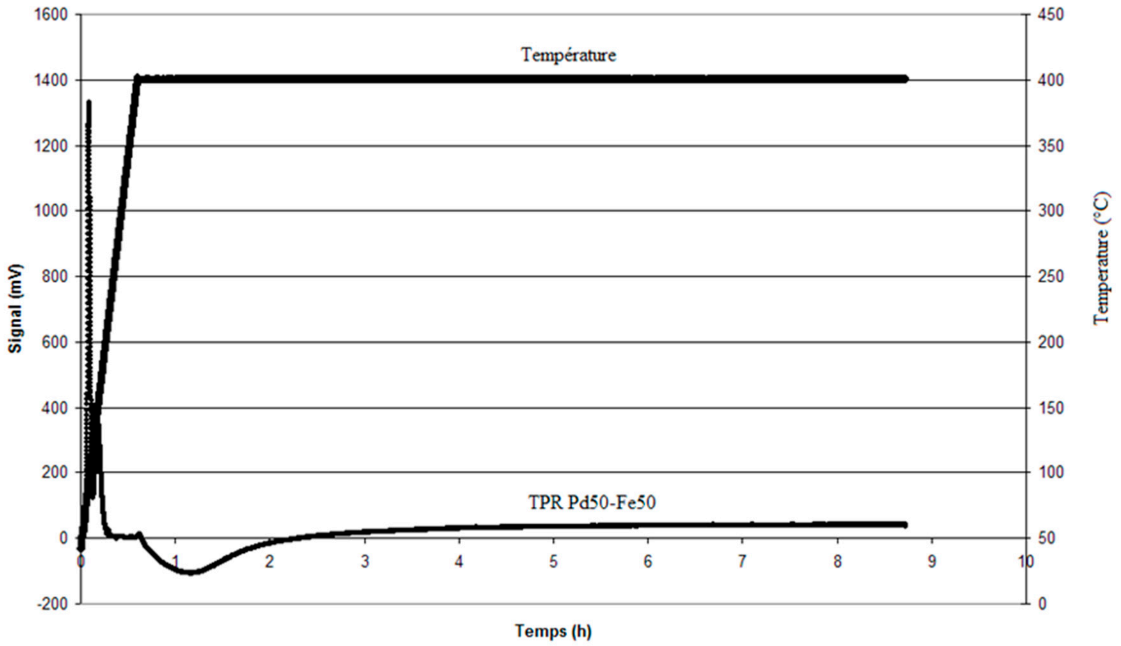

Figure S2. TPR of Pd50-Fe50 sample.
